# Single-Pass Albumin Dialysis (SPAD) as a Therapeutic Extracorporeal Liver Support in a Low-Resource Setting: Case Report and Literature Review

**DOI:** 10.7759/cureus.85782

**Published:** 2025-06-11

**Authors:** David Roman Hernández, Alicia Valdez-Gaona, Luis Arturo García-Velasco, Jordana S Lemus, Omar González Herrera, Guillermo Cardenas Membrila

**Affiliations:** 1 Intensive Care Unit, Hospital General “Dr. Manuel Gea Gonzalez”, Mexico City, MEX; 2 Intensive Care Unit, Hospital General “Dr. Manuel Gea Gonzalez", Mexico City, MEX; 3 Intensive Care Unit, Hospital General "Dr. Manuel Gea González", Mexico City, MEX; 4 Intensive Care Unit, Hospital General "Dr. Manuel Gea Gonzalez", Mexico City, MEX

**Keywords:** acute kidney injury (aki), acute liver failure (alf), acute-on-chronic liver failure (aclf), continuous venovenous hemodiafiltration (cvvhdf), intermittent hemodialysis (ihd), molecular adsorbent recirculating system (mars), single-pass albumin dialysis (spad)

## Abstract

Acute liver failure, a condition marked by high mortality, leads to a loss of liver function that impairs the detoxification of substances such as bilirubin, ammonia, and lactate, as well as the liver’s synthetic functions, causing damage to other organs. New extracorporeal therapies, including single-pass albumin dialysis (SPAD), have been developed to support liver function. We present a case of a 25-year-old male with no significant past medical history who presented with multiple organ failure and acute kidney injury that required continuous renal replacement therapy (CRRT) with continuous venovenous hemodiafiltration (CVVHDF), necessitating SPAD for the treatment of the liver failure.

## Introduction

The definition of liver dysfunction is still controversial, lacking a global consensus; liver failure is classified into two main entities based on the presence or absence of pre-existing liver disease [1].

It is defined as the loss of liver functions, such as the detoxification of substances (bilirubin, ammonia, glutamine, lactate, aromatic amino acids, free fatty acids, inflammatory cytokines), as well as the regulation and synthesis of albumin and coagulation factors, leading to a series of lethal complications, which frequently necessitates admission to the intensive care unit (ICU) [2].

Cirrhosis is a leading cause of mortality and morbidity across the world. It is the 11th leading cause of death and 15th leading cause of morbidity, accounting for 2.2% of deaths and 1.5% of disability‐adjusted life years worldwide in 2016. Chronic liver disease (CLD) caused 1.32 million deaths in 2017, approximately two‐thirds among men and one‐third among women [3].

Chronic liver failure ranks among the top five causes of death in Mexico and within the top 10 causes globally. Its main causes include alcohol abuse, hepatitis viruses, fatty liver disease, and autoimmune liver diseases. The term acute-on-chronic liver failure (ACLF) is defined as the acute deterioration of clinical conditions in patients with cirrhosis or pre-existing liver disease. Among patients with CLD admitted to the hospital, 24-40% will develop ACLF, which accounts for 1 to 5% of ICU admissions [4].

Acute liver failure (ALF) is a condition that presents with an abrupt onset, characterized by biochemical evidence of hepatocellular injury, coagulopathy, and encephalopathy. According to the interval between the appearance of jaundice and the onset of hepatic encephalopathy, O'Grady employed the terms hyperacute (< 7 days), acute (7-28 days), and subacute (4-12 weeks) [5]. Three scenarios can be identified according to the evolution and treatment: the first will improve with management and supportive measures, the second will require early liver transplantation, while the third will need support therapies used molecular adsorbent recirculating system (MARS), extracorporeal membrane oxygenation (ECMO), single-pass albumin dialysis (SPAD), continuous renal replacement therapy (CRRT)) as a bridge to clinical improvement or liver transplant. However, the benefits in terms of survival and mortality are still not clearly defined; these supportive modalities show a statistically favorable trend for this type of patient, demonstrating clinical improvement and better biochemical parameters [6].

There are low-income hospitals like ours that do not have therapies like MARS (Baxter) or Prometheus (Fresenius Medical Care), where less expensive therapeutic tools like SPAD could be used, which has been reported in some studies with similar efficacy [7].

## Case presentation

**Figure 1:**
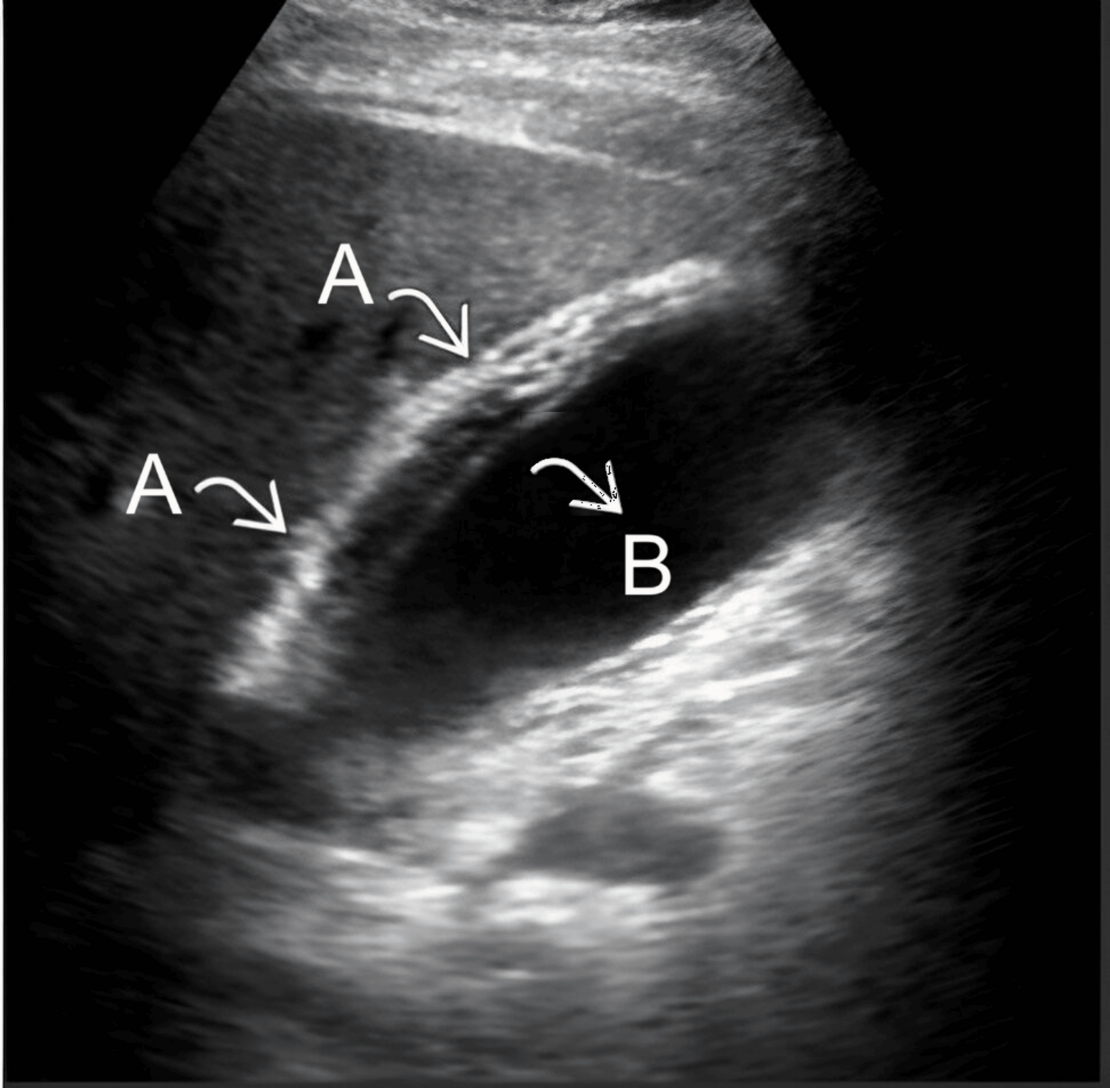
Ultrasound of the liver and biliary tract A) The gallbladder was found with edema, thickening, and stratification of the wall. B) Otherwise, gallbladder distention with absence of gallstones was observed.

A 25-year-old male patient with no significant personal medical history presented one week prior to admission with epigastric pain, generalized jaundice, dark urine, and pale stools. An ultrasound of the liver and biliary tract was performed, with a presumptive diagnosis of acalculous cholecystitis (Figure 1).

An endoscopic retrograde cholangiopancreatography (ERCP) was conducted, where obstructive jaundice was reported, leading to sphincterotomy. Following this, the patient experienced hematochezia, prompting admission to our hospital, where the following vital signs were recorded: BP 60/30 mmHg, HR 105 bpm, RR 35 breaths per minute. Treatment was initiated with crystalloids, with no clinical improvement, leading to the initiation of dual vasopressor support. The clinical analyses upon admission are reported in Table 1.

**Table 1:** Hospital Admission Laboratory Studies Data on kidney injury, as well as hyperbilirubinemia, were observed in laboratory studies.

Lab Test	Result	Reference Range	Lab Test	Result	Reference Range
Hemoglobin	8.75 g/dL	12.5-16.3 g/dL	Albumin	1.8 g/dL	4.2-5.5 g/dL
Hematocrit	26.54%	36.7-47.1%	ALT (Alanine aminotransferase)	63 U/L	7-52 U/L
Platelets	307000	152000-348000	AST (Aspartate aminotransferase)	86 U/L	13-39 U/L
Leukocytes	19000	4.0-12.0	GGT (Gamma glutamyl transferase)	102 U/L	9-64 U/L
Glucose	6.1 mmol/L	4.1-5.9 mmol/L	Amylase	1096 U/L	29-103 U/L
BUN (Urea nitrogen)	7.4 mmol/L	2.5-8.9 mmol/L	Lipase	1376 U/L	11-82 U/L
Creatinine	210.4 µmol/L	59.2-103.4 µmol/L	CPK (Creatine phosphokinase)	286 U/L	30-233 U/L
PT (Prothrombin time)	20 Sec	9.4-12.5 Sec	Myoglobin	26.8 µmol/L	0-4.1 µmol/L
INR (International Normalized Ratio)	1.82	0.80-1.20	Ph	7.26	7.35-7.45
aPTT (Activated partial thromboplastin time)	28 Sec	23.0-40.0 Sec	pCO_2_ (partial pressure of carbon dioxide)	46 mmHg	35.0-48.0 mmHg
Total bilirubin	160.7 µmol/L	5.1-17.1 µmol/L	pO_2_ (partial pressure of oxygen)	36 mmHg	83.0-105.0 mmHg
Direct bilirubin	110 µmol/L	0.5-3.1 µmol/L	Lactate	6.7 mmol/L	0.38-0.75 mmol/L
Indirect bilirubin	52.2 µmol/L	4.6-14 µmol/L	EB (base excess)	-11.5	2-3
Alkaline phosphatase	80 U/L	34-104 U/L			

**Figure 2:**
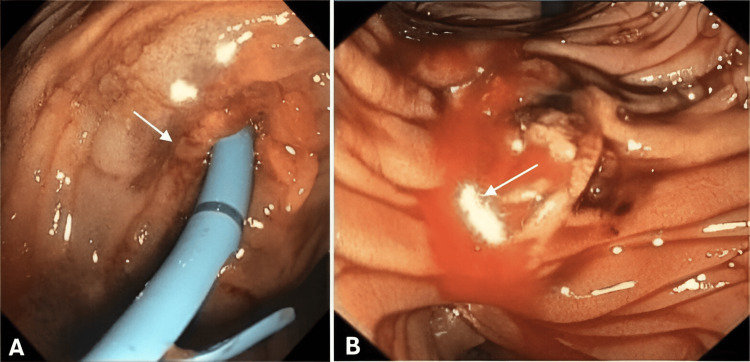
Endoscopic retrograde cholangiopancreatography (ERCP) ERCP observing bleeding (A) and discharge of purulent material (B) from sphincterotomy.

The patient experienced further hemodynamic instability, multiple organ failure, and rhabdomyolysis, along with a decrease in hemoglobin; therefore, a transfusion of six units of fresh frozen plasma and five red blood cell concentrates was indicated. Additionally, neurological and respiratory deterioration was noted, prompting the initiation of advanced airway management. A new ERCP was performed with the placement of a biliary stent, observing the discharge of purulent material as well as bleeding from the previously performed sphincterotomy (Figure 2). Antimicrobial treatment with meropenem was started.

The patient was admitted to the ICU, where the following values were found (Table 2). Serologies for hepatitis A, B, and C viruses were non-reactive.

**Table 2:** Intensive Care Unit Admission Laboratory Studies Despite the treatment, greater deterioration of kidney and liver function was observed.

Lab Test	Result	Reference Range	Lab Test	Result	Reference Range
Hemoglobin	7.47 g/dL	12.5 to 16.3 g/dL	Indirect bilirubin	43.4 µmol/L	4.6-14.0 µmol/L
Leukocytes	9200	4.0-12.0	AST (Aspartate aminotransferase)	1348 U/L	13-39 U/L
Platelets	73000	152000-348000	ALT (Alanine aminotransferase)	2974 U/L	7-52 U/L
Total bilirubin	144 µmol/L	5.1-17.1 µmol/L	CPK (Creatine phosphokinase)	25567 U/L	30-223 U/L
Direct bilirubin	100.04 µmol/L	0.5-3.1 µmol/L	Creatinine	465 µmol/L	59.2-103.4 µmol/L

**Figure 3:**
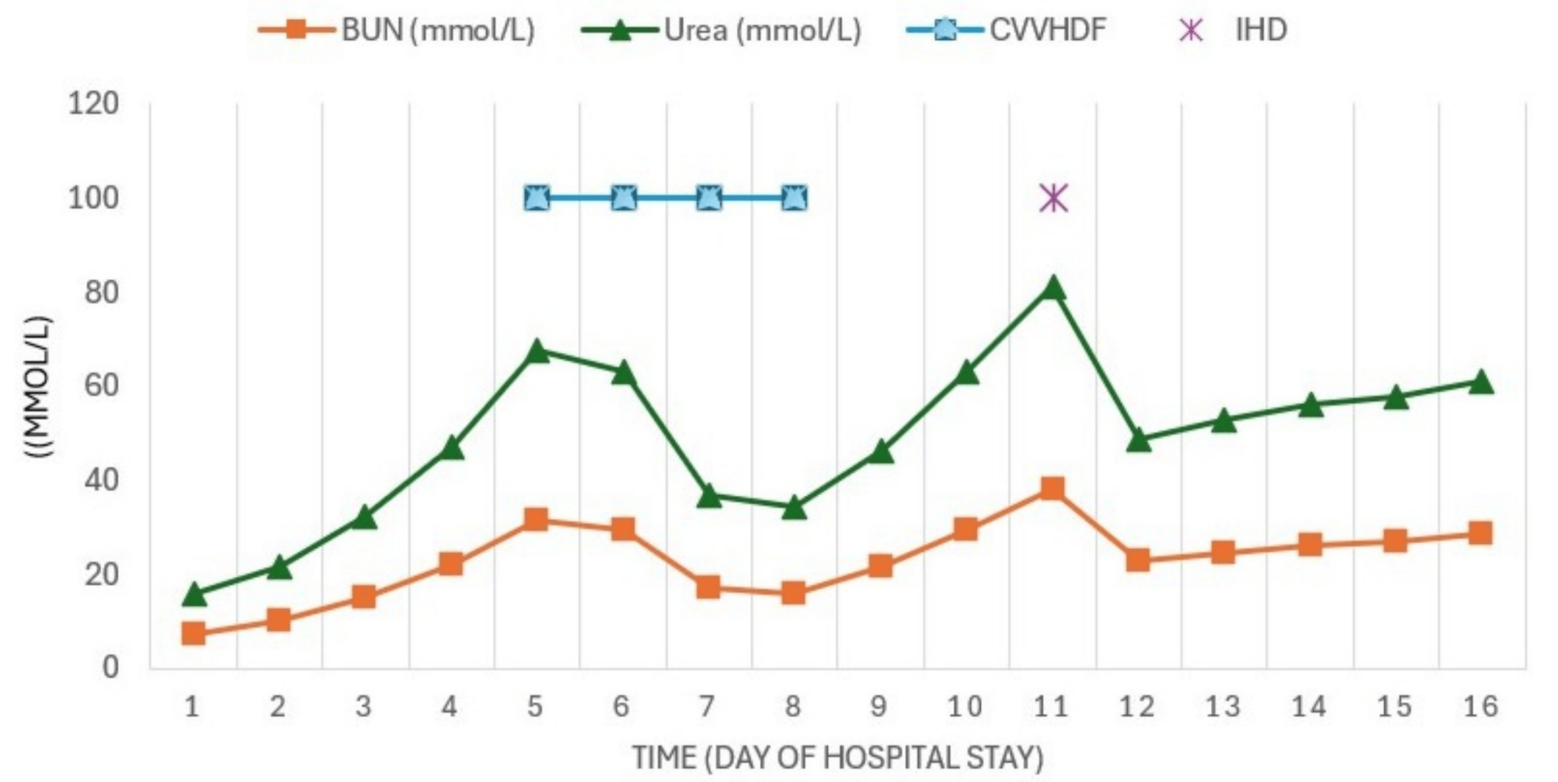
Evolution of azotemia The figure shows the behavior of the azotemia and how it decreased with CVVHDF days later, IHD was performed. CVVHDF: continuous venovenous hemodiafiltration; IHD: intermittent hemodialysis

Due to progressive elevation of nitrogenous waste, metabolic acidosis, and oliguria (acute kidney injury (AKI) KDIGO 3), continuous renal replacement therapy (CRRT) was initiated using continuous venovenous hemodiafiltration (CVVHDF) with an effluent dose of 30 ml/kg/h and a filtration fraction (FF) of 22% (Figure 3).

**Figure 4:**
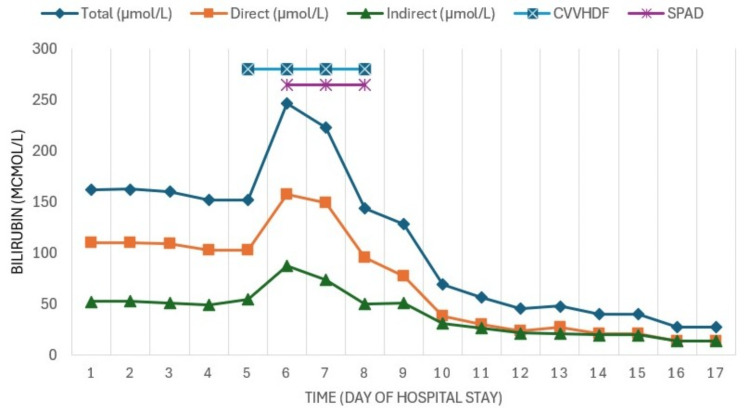
Evolution of bilirubin tests Since hyperbilirubinemia and liver failure persisted despite stent placement by ERCP, SPAD therapy was started, which achieved a significant reduction in bilirubin.

**Figure 5:**
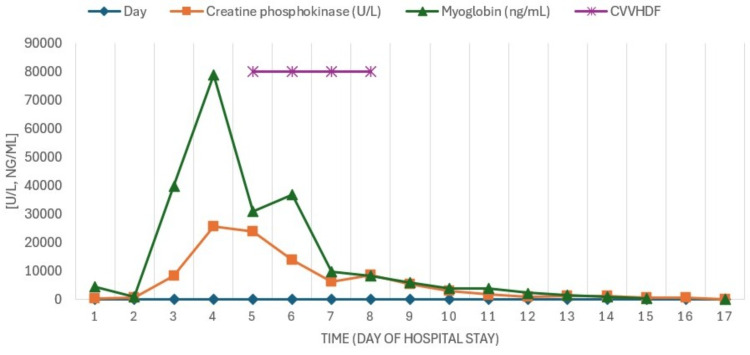
Evolution of muscle enzymes With CVVHDF, an effective decrease in serum myoglobin levels with improvement in rhabdomyolysis was also observed. CVVHDF: continuous venovenous hemodiafiltration

**Figure 6:**
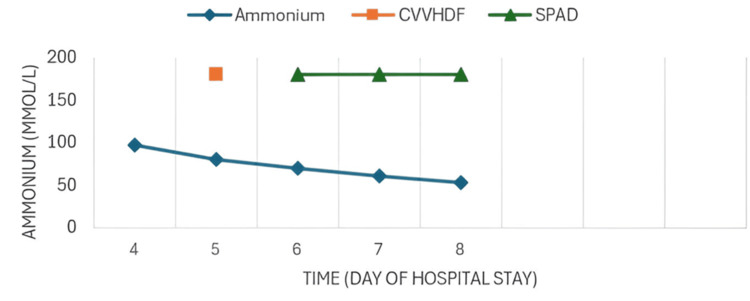
Evolution of determination of ammonium With SPAD and CVVHDF, an effective decrease in serum ammonium levels with improvement was observed. CVVHDF: continuous venovenous hemodiafiltration; SPAD: single-pass albumin dialysis

As the patient experienced worsening hepatic failure, SPAD was added to the CRRT, utilizing 3% albumin. Three sessions were conducted every 24 hours, each lasting six hours, with a dialysate flow rate (Qd) of 1000 ml/h, resulting in clinical and biochemical improvement with decreases in azotemia (Figure 3), bilirubin (Figure 4), CPK, myoglobin (Figure 5), and ammonia (Figure 6) as well as recovery of renal function. 

**Figure 7:**
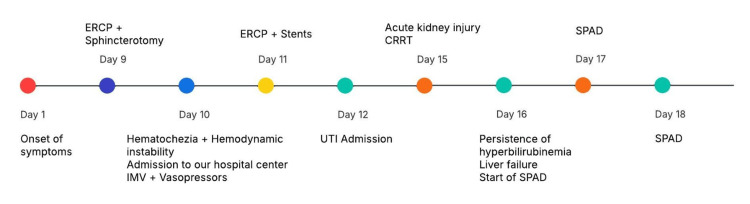
Timeline of clinical progression Shown from the day of symptom onset to the initiation of SPAD therapy. SPAD: single-pass albumin dialysis

The timeline of clinical progression is shown in Figure 7. Respiratory improvement was also noted, leading to the decision for extubation. The patient was monitored for 48 hours and was discharged from the service after a 10-day stay in the ICU.

## Discussion

Acute acalculous cholecystitis manifested by the presence of an inflammation of the gallbladder in the absence of stones inside, it is characterized by presenting clinical manifestations that do not differ greatly from those of lithiasic cholecystitis, among them; fever, jaundice, localized pain in the right hypochondrium, nausea, vomiting and anorexia, positive or doubtful Murphy sign, hypersensitivity of the area and presence of mass in upper quadrant, corresponding to this, should be considered among the diagnostic possibilities for every patient critically ill or injured with a clinical presentation of sepsis or jaundice with no known origin [8].

ALF should be considered a medical emergency due to its rapidly progressive nature, multi-organ involvement, and high risk of mortality, necessitating early diagnosis and timely, etiological, multidisciplinary treatment, including supportive therapies such as liver transplantation. Hepatocellular injury has various etiologies; it can be caused by states of sepsis, drug toxicity, neoplasms, herbal treatments, or as a consequence of the failure of other organs. In these situations, it is essential to address the underlying etiology and initiate measures to prevent progression. Although the pathophysiology is not yet fully understood, once damage is initiated by an acute decompensation, an inflammatory response is triggered both locally and systemically, often leading to multiple organ failure [9].

The prognosis for these patients will depend on the cause and severity, with a mortality rate of up to 80%, and low survival rates if liver transplantation is not performed. Due to the absence of a specific treatment, timely diagnosis and management, as well as decision-making, are of vital importance. The therapeutic approach consists of supportive measures, addressing the triggering factor, managing complications, and timely evaluation for liver transplantation, as this represents the definitive treatment for acute-on-chronic liver failure [10].

The liver loses metabolic functions and protein synthesis in both acute and chronic liver failure, in addition to the release of inflammatory mediators, which contributes to multi-organ dysfunction. Since 1990, several extracorporeal liver support systems have been described, classified as biological and non-biological. The former are based on the use of cells (hepatocytes) from humans or animals, which function to supplement the hepatocyte; the latter utilize therapeutic modalities characterized by the removal of toxic substances through high-volume apheresis, dialysis, or adsorption [11].

Extracorporeal liver support therapies aim to serve as a bridge to liver transplantation or to recovery from ALF. These modalities are still under development; on one hand, few studies have been published, and on the other, the data obtained have been controversial, as they do not show significant differences in survival prognosis, with liver transplantation being the only therapy that has demonstrated an impact on mortality reduction, both in ALF and ACLF. Intermittent Hemodialysis (IHD) is a widely known renal replacement therapy modality, applicable to patients with both acute and chronic kidney disease. Its mechanisms of action include diffusion and convection; the former refers to the passage of solutes through a semipermeable membrane by a concentration gradient, while the latter is based on the passage of solutes through a pressure gradient (drag mechanism). In patients with hepatic failure, whether acute or chronic, associated with renal damage, the use of slow therapies is recommended, as these are better tolerated in patients with hemodynamic instability compared to IHD [11]. There is a limitation in the clearance of other types of molecules, as only water-soluble solutes are extracted and not those bound to albumin. Hepatic toxins are often hydrophobic with a molecular weight of <1000 Da (bilirubin = 406 Da, cholic acid = 283 Da, chenodeoxycholic acid = 272 Da, tryptophan = 204 Da, phenol = 94 Da, etc.) [12].

Conventional dialysis therapies in patients with hepatic failure are insufficient, involving the use of modalities whose mechanism allows for the extraction of hydrophobic and protein-bound solutes such as albumin. Among the hepatic support modalities, plasmapheresis and hemoperfusion were initially described; the former operates by the direct extraction of plasma, achieving detoxification, while the latter is based on the adsorption of lipid-soluble solutes from the blood through an activated carbon filter. These therapies have demonstrated relative efficacy but come with significant complications such as infections, bleeding, and thrombocytopenia, which contribute to greater hemodynamic instability. Recently, other techniques such as MARS and SPAD have been developed, which have shown a clinically greater benefit in hemodynamically unstable patients; however, the actual benefit in survival remains in question [13].

MARS is a detoxification therapy that allows for the semi-elective removal of albumin-bound toxins using a standard hemodiafiltration machine, to which a module is added that enables the adaptation of an intermediate circuit with human albumin at concentrations of 10-20%. This circuit allows for facilitated diffusion of substances bound to albumin, thus combining conventional hemodialysis, capable of removing hydrophilic substances (with a molecular weight of less than 50 kDa), and dialysis with albumin to eliminate hydrophobic substances bound to it (albumin has a molecular weight of approximately 67 kDa). Thus, the bound and free substances diffuse through the membrane, with the former binding to exogenous albumin. The dialysate forms the component of the second circuit that subsequently passes through the blood compartment of a conventional dialysis filter, which removes hydrophilic substances, and through two cartridges (one of resin and another of activated carbon) that extract those bound to albumin. The use of this technique can be intermittent at intervals of 6 to 8 hours daily or continuous, and the flow rate will depend on the hemodynamic status of the patient [13].

**Figure 8:**
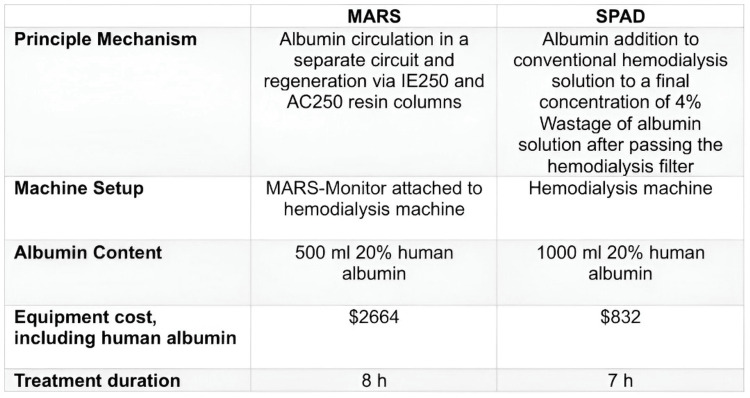
Comparison between MARS vs SPAD SPAD: single-pass albumin dialysis; MARS: molecular adsorbent recirculating system Comparison between MARS vs SPAD.

In terms of removal capacity, MARS eliminates hepatic toxic substances such as bilirubin, bile acids, aromatic amino acids, phenol, mercaptan, lactate, glutamine, oxidative stress mediators, fatty acids, pro-inflammatory cytokines, and vasoactive mediators; this technique has been used in patients with ACLF, IHA, post-transplant liver failure, drug intoxication, and liver dysfunction secondary to critical illness. The benefit regarding survival is still controversial, as no significant differences have been demonstrated; most studies have included few patients, so the results should be interpreted with caution. Nevertheless, these types of therapies are a promising alternative in the field of hepatic detoxification, with frequent limitations for their use being limited accessibility, complexity, and high cost (Figure 8) [14].

**Figure 9:**
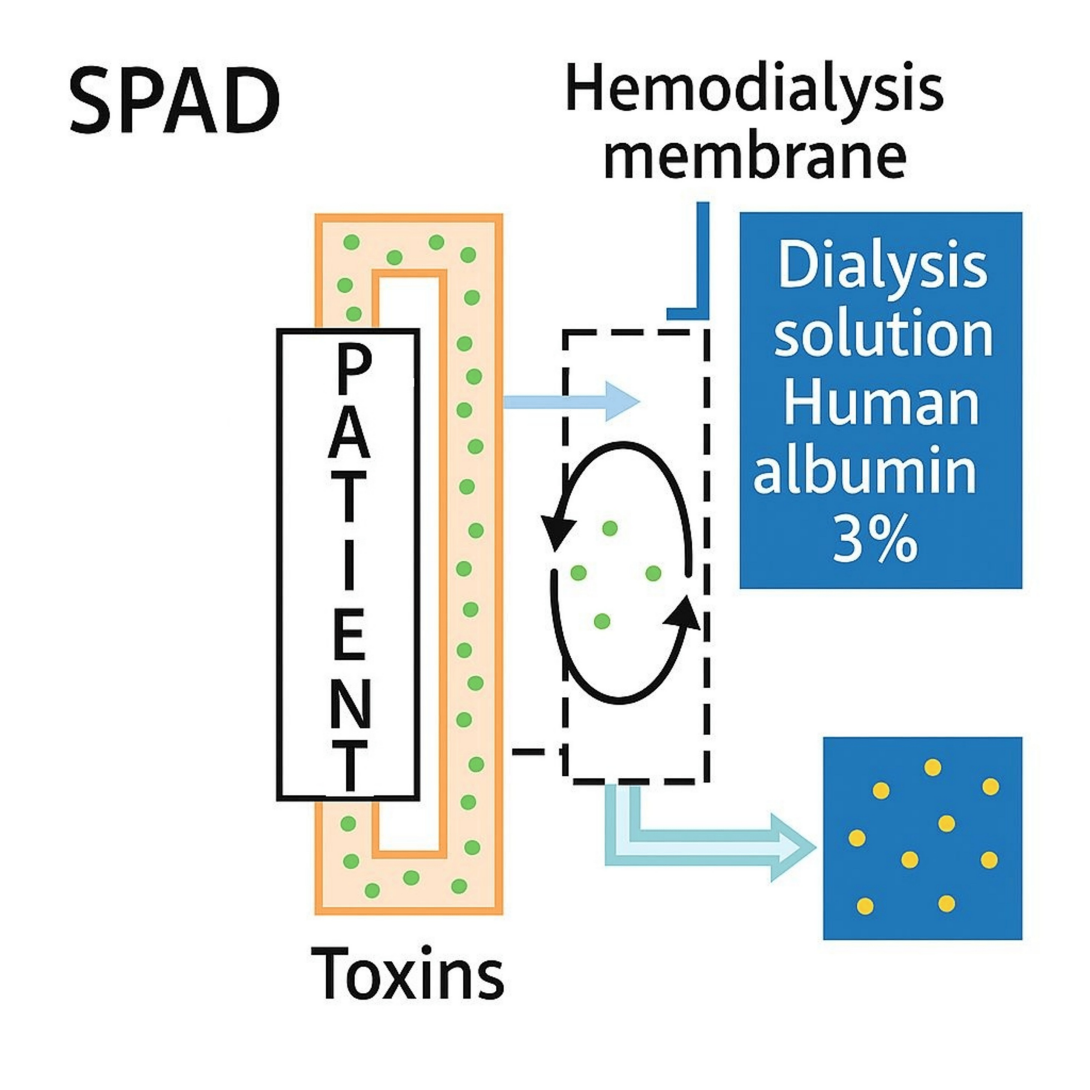
Single-pass albumin dialysis (SPAD) For SPAD (PrismaFlex, Baxter), 750 ml of fluid was removed from a 5000 ml dialysis solution bag (PrismaSate dialysate for heparin anti-coagulation) and replaced with 15 bottles of 20% human albumin solution (50 ml each) to get an albumin concentration of 3%. Using a 5000 ml dialysis solution bag, the flow rate of 1000 ml/h resulted in a treatment cycle of about 6 hours. SPAD: single-pass albumin dialysis

SPAD is one of the techniques used in extracorporeal liver support therapy as well as a bridge to recovery of function, first reported in 1999 by Siege et al.; this procedure involves the passage of the patient's blood through a high-flow membrane that is simultaneously counter-current to a dialysis fluid enriched with albumin. This technique can be used in isolation or combined with a filter to perform CVVHDF. The SPAD therapy, compared to MARS, has the advantage of lower complexity as it utilizes conventional dialysis equipment and does not require any additional modules (Figure 9) [15]. This process generates a high consumption of albumin, as it is removed along with other toxic substances, which increases its cost; however, this cost decreases when using 3-5% dilutions (unlike MARS, which uses 20% albumin). Furthermore, this system does not require an interposed circuit with adsorbent cartridges, as used in MARS, since albumin is not recycled [16].

Two studies compared the costs and efficacy of SPAD with respect to MARS, both agreeing that the former therapy could be more effective in eliminating certain substances, such as bilirubin, bile acids, and urea, while being less effective in clearing molecules like creatinine and ammonium. However, few studies have demonstrated a statistically significant benefit in survival [14,17,18].

In the case we describe, due to the patient presenting with AKI KDIGO 3 and hemodynamic instability, CRRT was initially utilized in the CVVHDF modality, taking advantage of the solute removal characteristics that this modality offers to reduce elevated serum myoglobin levels. However, upon observing further deterioration of liver function and lacking MARS in our hospital, it was decided to add SPAD to the treatment as an alternative form of extracorporeal support therapy, which demonstrated a clear benefit in terms of reducing hepatic toxins (bilirubin and ammonia), uremic toxins, correcting acidosis, and decreasing muscle enzymes (CPK, myoglobin) [19,20].

After three sessions of SPAD, the concentrations of the aforementioned toxins gradually decreased until normalization, also achieving recovery of renal function and discharge from the ICU.

## Conclusions

SPAD is a modality of extracorporeal liver therapy that has been gaining popularity, it has therapeutic objectives similar to MARS, but with the advantage of not requiring a specialized module for its application and using albumin in lower concentrations, which translates to lower costs. This allows for the reduction of toxic substances, both hepatic (mainly bilirubin and ammonia) and extrahepatic (CPK, myoglobin, creatinine, urea, and BUN), leading to clinical improvement and better biochemical parameters in the patient.

Furthermore, there is still no consensus regarding the timing of initiation, the duration of each session, or the number of sessions. As this type of therapy is not yet widely disseminated, it needs to be evaluated and validated in prospective studies to gain greater clarity regarding its benefits and risks. Therefore, it is essential to increase experience in its prescription, which could lead to greater benefits for the patient, while keeping in mind that transfer to a hospital capable of performing liver transplantation should not be delayed if indicated.
